# Cu/Zn-superoxide dismutase forms fibrillar hydrogels in a pH-dependent manner via a water-rich extended intermediate state

**DOI:** 10.1371/journal.pone.0205090

**Published:** 2018-10-05

**Authors:** Noriko Fujiwara, Michiru Wagatsuma, Naoto Oba, Daisaku Yoshihara, Eiichi Tokuda, Haruhiko Sakiyama, Hironobu Eguchi, Motoko Ichihashi, Yoshiaki Furukawa, Tadashi Inoue, Keiichiro Suzuki

**Affiliations:** 1 Department of Biochemistry, Hyogo College of Medicine, Nishinomiya, Hyogo, Japan; 2 Research and Development, ULVAC, Chigasaki, Kanagawa, Japan; 3 Department of Macromolecular Science, Graduate School of Science, Osaka University, Toyonaka, Osaka, Japan; 4 Department of Chemistry, Keio University, Yokohama, Kanagawa, Japan; University of South Carolina, UNITED STATES

## Abstract

Under certain conditions, amyloid-like fibrils can develop into three-dimensional networks and form hydrogels by a self-assembly process. When Cu/Zn superoxide dismutase (SOD1), an anti-oxidative enzyme, undergoes misfolding, fibrillar aggregates are formed, which are a hallmark of a certain form of familial amyotrophic lateral sclerosis (ALS). However, the issue of whether SOD1 fibrils can be assembled into hydrogels remains to be tested. Here, we show that the SOD1 polypeptides undergo hydrogelation accompanied by the formation of thioflavin T-positive fibrils at pH 3.0 and 4.0, but not at pH 5.0 where precipitates are formed. The results of viscoelastic analyses indicate that the properties of SOD1 hydrogels (2%) were similar to and slightly more fragile than a 0.25% agarose gel. In addition, monitoring by a quartz crystal microbalance with admittance analysis showed that the denaturing of immobilized SOD1 on a sensor under the hydrogelation conditions at pH 3.0 and 4.0 resulted in an increase in the effective acoustic thickness from ~3.3 nm (a folded rigid form) to ~50 and ~100 nm (an extended water-rich state), respectively. In contrast, when SOD1 was denatured under the same conditions at pH 5.0, a compact water-poor state with an effective acoustic thickness of ~10 nm was formed. The addition of physiological concentrations of NaCl to the pH 4.0 sample induced a further extension of the SOD1 with larger amounts of water molecules (with an effective acoustic thickness of ~200 nm) but suppressed hydrogel formation. These results suggest that different denatured intermediate states of the protein before self-assembly play a major role in determining the characteristics of the resulting aggregates and that a conformational change to a suitable level of extended water-rich intermediate state before and/or during intermolecular assembling is required for fibrillation and hydrogelation in the case of globular proteins.

## Introduction

Fibrillation is considered to be an innate property that is common to all polypeptides, and amyloid-like fibrillar aggregates have been found in a number of neurodegenerative diseases, including Alzheimer’s disease, Parkinson’s disease, prion diseases and amyotrophic lateral sclerosis (ALS). Under certain conditions, fibrous proteins such as gelatin [1] and fibrils derived from a specific peptide motif such as elastin-like pentapeptides, VPGXG (where X is any residue but proline) [2] and Ac-C(FKFE)_2_CG-NH_2_ [3] can develop into three-dimensional networks and form hydrogels by self-assembly. Amyloidogenic peptides, such as the β-amyloid diphenylalanine [4], islet amyloid polypeptide [5], were also reported to produce viscoelastic hydrogels consisting of fibrillar meshworks. Furthermore, globular proteins, such as insulin [6], β-lactoglobulin [7, 8], bovine serum albumin (BSA) [9] and α-synuclein [10] also are known to form hydrogels. Intriguingly, fused in sarcoma (FUS), one of the proteins that causes ALS also forms fibrillar hydrogel-like assemblies that impair the function of ribonucleoprotein granules [11, 12]. Hydrogelation is, therefore, thought to be another important property that is inherently registered in polypeptides. Furthermore, it has been proposed that the primary cause of cell death in amyloid neurodegenerative diseases is the physical effect of the amyloid gels and not chemical toxicity, since amyloid gels would halt the convection essential to the transport of dissolved molecules in and out of cells [13]. Therefore, an understanding of the mechanism responsible for the formation of protein gels becomes important. However, the mechanism is still unclear as to whether the amyloid-like fibrils proceeds to hydrogelation or proceeds to aggregation.

Cu/Zn-superoxide dismutase (SOD1) is a primary anti-oxidative enzyme that catalyzes the conversion of superoxide anion radicals into less reactive hydrogen peroxide and molecular oxygen. On the other hand, SOD1 is known to be an ALS-causative protein, and the aggregation of mutant SOD1 is a pathological hallmark in familial forms of ALS with mutations in the *SOD1* gene [14–16]. Although SOD1 knockout mice show some impairments [17, 18], they do not develop ALS-like symptoms [19]. SOD1 is a homodimer containing one copper ion required for enzymatic activity and one zinc ion required for the protein stability in each 16-kDa subunit. While fully metallated human SOD1 is a quite stable globular protein melting at 94°C in differential scanning calorimetry experiments [20], ALS-linked mutant SOD1 proteins are significantly less stable [21, 22] and prone to misfolding and aggregation [22–24]. The reduction of the intrasubunit disulfide bond (Cys57-Cys146) and loss of metals in both mutant and wild-type SOD1 results in monomerization, followed by the formation of amyloid-like fibrillar aggregates [25–27]. Thioflavin-S-positive fibrillar aggregates composed of at least partly of SOD1 have also been characterized in neural tissues of ALS-model mice [28]. Nonetheless, it remains unknown whether SOD1 fibrils are further self-assembled into hydrogels.

Quartz crystal microbalance (QCM) techniques have been developed for obtaining, not only changes in mass but also the viscoelastic properties of molecules. Generally, QCM detects the mass of materials that had been adsorbed to the electrode surface as a frequency change, which is related to mass uptake via the Sauerbrey equation [29]. However, if the adsorbed layer (adlayer) on a sensor is not rigid but viscoelastic, the frequency change will not fit the Sauerbrey equation as described in the Supporting information (S1 File). QCM-D method, which is more widely used, typically senses the energy dissipation value (*D*-value) and the effective acoustic thicknesses when the crystal oscillation decays using a flow-cell system. QCM-D monitors viscoelastic changes accompanying multilayer amyloid deposition [30, 31] and conformational changes in the adsorbed protein monolayer [32]. An alternate method is the QCM technique, based on admittance (QCM-A) analysis, which obtains the admittance from the resonance frequency by using equipment such as a network analyzer. Because the QCM-A used in this study utilizes a batch cell system, the solutions in a vessel can be easily changed. QCM-A as well as QCM-D can be used to obtain an adsorbed mass, effective acoustic thicknesses and viscoelasticity such as the *D*-value of the sample adlayer on the sensor surface.

In this study, we report that an ALS-causing stable globular protein, SOD1, undergoes hydrogelation or aggregation accompanied by the formation of amyloid-like fibrils and that these conversions are pH-dependent. Monitoring of the immobilized SOD1 molecules with QCM-A permitted the different properties between water-rich extended molecules to be distinguished, under fibrillation and hydrogelation conditions (pH 3.0 −4.0) and water-poor compact molecules under precipitation conditions (pH 5.0).

## Materials and methods

### Reagents and proteins

All chemicals used in this study were purchased either from Wako Pure Chemical Industries Ltd. or Sigma-Aldrich, unless specified otherwise, and were of the highest grade available. Recombinant wild-type human SOD1 (residues 1–153) modified by disulfide bonding with 2-mercaptoethanol (2-ME) at Cys^111^ (Cys111-S-S-CH_2_CH_2_OH) was generously donated by Ube Industries Ltd. The fact that 2-ME only modified the side chain of Cys111, and not Cys6, was verified by MALDI–TOF MS (matrix-assisted laser-desorption ionization–time-of-flight MS) analysis [33]. This 2-ME-SOD1 is stable in solution (100 mg/mL) and is easily converted back to the wild-type SOD1 by reduction with 20 mM 2-ME [33, 34]. Gelatin solution was purchased from Nitta gelatin.

### Thioflavin T fluorescence assay

SOD1 fibrillation was monitored by following the increase in the fluorescence intensity of thioflavin T (ThT). Two hundred μL of sample solutions containing various concentrations of SOD1, with or without DTT, EDTA and 20 μM ThT in buffers at various pH values in a 96-well plate were incubated at 37°C, and the ThT fluorescence intensity was recorded at 2 min intervals using a plate reader (Tecan Infinite M200 PRO) with excitation and emission wavelengths of 450 and 485 nm, respectively. The plate was shaken for 1 min at 200 rpm prior to each fluorescence reading.

### Transmission electron microscopy

SOD1 hydrogels or SOD1 aggregates were absorbed on STEM100Cu elastic carbon coated grids (Okensyoji, Japan), washed with water and then negatively stained with 2% Samarium (III) acetate hydrate. Images were obtained using an electron microscope (Tecnai Spirit, FEI).

### Hydrogel formation in centrifuge tubes or glass vials

2ME-SOD1 (final concentration: 20 mg/mL) was incubated overnight with 10 mM EDTA and 100 mM dithiothreitol (DTT) in a 50 mM acetate buffer (pH 3.0−5.0) or phosphate buffer (pH 6.0−8.0) at 37°C without shaking, which is a similar method for producing monomeric SOD1 [35].

### Rheology

Rheological measurements were carried out using a stress-controlled rheometer (MCR 302 Modular Compact Rheometer; Anton Paar Japan, K.K., Tokyo) with a cone-plate geometry (diameter *d* = 40 mm, cone angle *θ* = 0.7 rad) equipped with a solvent trap filled with water to prevent evaporation. The SOD1 hydrogels were transferred to the stage and the frequency dependences of the complex moduli (*Gʹ* and *Gʹʹ*) were recorded under a constant shear strain of 2% maintained over the angular frequency range of 0.1 to 100 rad/s at 37°C. The amplitude dependences of the complex moduli were measured from 0.1% to 10% shear strain with an angular frequency of 20 rad/s at 37°C to investigate fragility of the hydrogels. When the network in the gel is broken by large amplitude strain, the complex modulus would change. For measurements of viscoelastic properties of the initial hydrogelation of SOD1 in a 50 mM an acetate buffer (pH 3.0–5.0) containing 10 mM EDTA and 100 mM DTT, rheological parameters, *Gʹ* and *Gʹʹ*, were monitored at 1 min intervals with an angular frequency of 20 rad/s at 37°C with 5% shear strain.

### Quartz crystal microbalance based on admittance analysis

A 27 MHz QCM device, AFFINIX QN Pro (ULVAC, Japan), based on admittance (QCM-A) analysis [36] was used in the current study. A detailed explanation of QCM-A can be found in S1 File and S1 Fig. For chemically immobilizing of native SOD1 by amine coupling on the sensor, an immobilizing protein kit was used according to the manufactory protocol. For direct immobilizing, a 1.0 mg/mL solution of native SOD1 or other proteins, such as gelatin, myosin, IgG, BSA, in 0.5 mL of 50 mM acetate buffers (pH 4.0) was added to the reaction vessel, followed by oscillating at 37°C with stirring (1,000 rpm) to saturate the adsorption. The native SOD1 was adsorbed at a level of ~350 ng/cm^2^. After immobilizing the protein, the vessel solution was changed to fresh acetate buffers (pH 3.0 to 5.0) containing 10 mM EDTA and 200 mM DTT, followed by starting the measurement at 37°C with stirring (1,000 rpm) and the frequency parameters were recorded at 2 sec intervals. After monitoring, effective acoustic thickness, mass, *D*-value and shear viscosity were calculated.

## Results and discussion

### High concentrations of SOD1 under fibrillation conditions formed hydrogels

**Fig 1:**
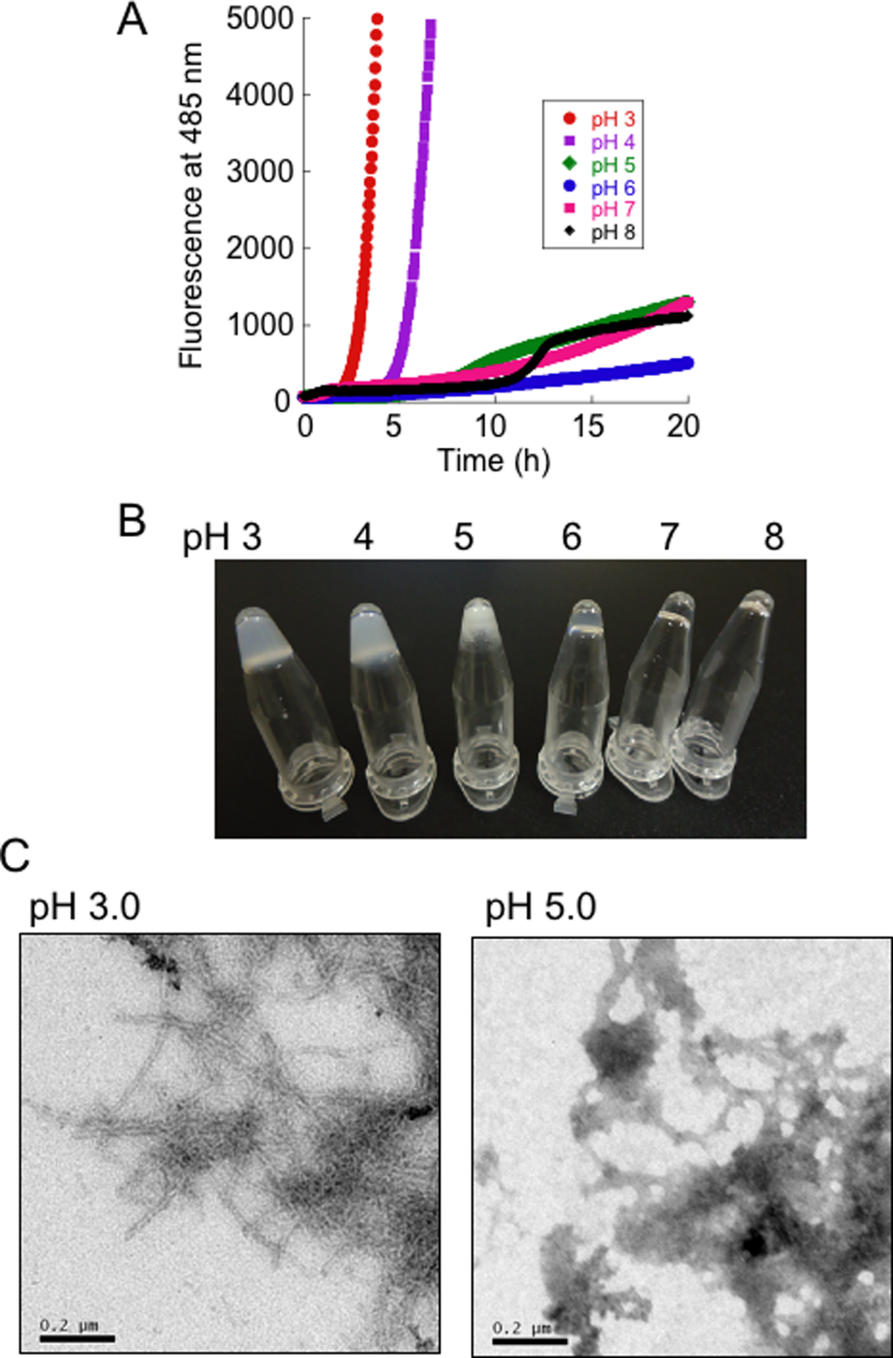
Fibrillation and hydrogelation of SOD1. **(A)** Fibrillation kinetics of SOD1 solutions (5 mg/mL) under fibrillation conditions in buffers at various pH values, as monitored by thioflavin T fluorescence. Representative kinetic data of three ThT assays are shown. (**B**) Images of micro-tube inversion tests for the SOD1 solutions (20 mg/mL) in buffers at various pH values under fibrillation conditions without shaking. **(C)** Transmission electron microscopy (TEM) images of samples processed at pH 3.0 and 5.0.

Reduction of the intrasubunit disulfide bond (Cys57-Cys146) and a loss of metals lead to the fibrillation of human SOD1 [26]. Thus, to investigate the effects of pH on the fibrillation of SOD1, a ThT assay was performed. When a 5 mg/mL solution of 2ME-SOD1 was allowed to shake with 100 mM DTT and 10 mM EDTA in buffers at various pH values, between pH 3.0 to pH 8.0 with 20 μM ThT at 37°C, the fastest increase in ThT fluorescence intensity was observed at pH 3.0, and the second fastest was pH 4.0. At pH values higher than pH 5.0, a much longer time was required to achieve fibrillation (Fig 1A). We confirmed that the Cys57-Cys146 disulfide bonds were completely reduced after a 24 h treatment with 100 mM DTT and 10 mM EDTA, even in acidic buffers (data not shown). After the ThT assay, we noted that the SOD1 fibril solutions at pH 3.0 and 4.0 had changed to a jelly-like solid. Thus, higher concentrations of SOD1 (20 mg/mL) were incubated with 100 mM DTT and 10 mM EDTA in the buffers (pH 3.0 to 8.0) at 37°C for 20 h without shaking. Fig 1B shows the state of the SOD1 samples in the microtubes after being inverted and tapped with a finger after the incubation. Intriguingly, the SOD1 samples in the pH 3.0 and pH 4.0 buffers became solid like hydrogels. In contrast, the SOD1 sample at pH 5.0 formed a precipitate and the supernatant ran down the side of the tube. The SOD1 samples at pH 6.0, 7.0 and 8.0 did not form any insoluble materials and behaved as solutions. We confirmed by size-exclusion chromatography that lower concentrations of SOD1 (1 mg/mL) were completely monomerized under the same conditions in all the buffers (pH 3.0 to 8.0) (data not shown). In addition, electron microscopy observations showed that the hydrogels in the pH 3.0 buffer exhibited a fibrous morphology but the precipitates in the pH 5.0 buffer exhibited an amorphous-like morphology (Fig 1C).

### Viscoelastic properties of SOD1 hydrogels analyzed by a rheometer

**Fig 2:**
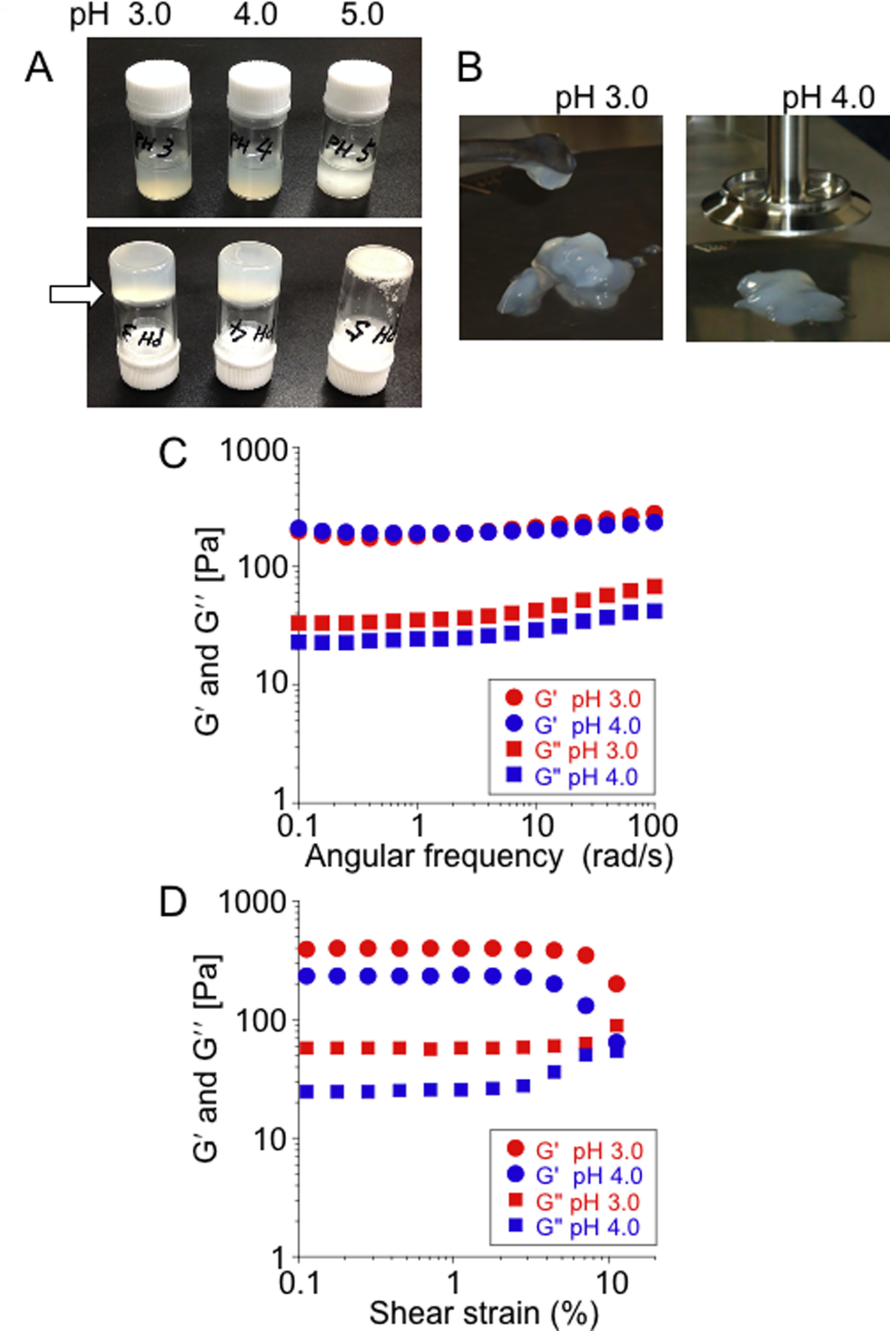
Rheological measurements of SOD1 hydrogel. **(A)** Images of vial inversion tests for SOD1 solutions (20 mg/mL) at pH 3.0, 4.0 and 5.0 after incubation at 37°C for 16 h under fibrillation conditions; upright vial bottle (upper panel) and inverted vial bottle (lower panel). (**B**) An image of scooped pH 3.0 and pH 4.0 gels. **(C)** Shear storage moduli (*Gʹ*) and shear loss moduli (*Gʹʹ*) of hydrogels derived from the pH 3.0 and pH 4.0 vials measured at 37°C with a 2% shear strain. (**D**) Strain dependence of *Gʹ* and *Gʹʹ* of hydrogels derived from the pH 3.0 and pH 4.0 vials measured at 37°C with the angular frequency of 20 rad/s.

To visualize the hydrogelation more clearly, 2 mL aliquots of SOD1 solutions (20 mg/mL) were denatured in glass vials under fibrillation conditions in pH 3.0, 4.0 and 5.0 buffers. As shown in Fig 2A, the vial inversion test reproduced the results shown in Fig 1B. Both hydrogels in the pH 3.0 and pH 4.0 vials were completely solidified and the SOD1 sample at pH 5.0 formed a precipitate. The structures of hydrogels in the pH 3.0 and pH 4.0 vials were destroyed when they were disrupted with a spatula (Fig 2B). The SOD1 hydrogels were then transferred to the stage of a rheometer and their viscoelastic properties were evaluated. The storage moduli (*Gʹ*) and the loss moduli (*Gʹʹ*) of both hydrogels reached ~200 Pa and 20−30 Pa, respectively (Fig 2C). When the elastic (*Gʹ*) response is superior to the viscous (*Gʹʹ*) one at the low frequency limit, *Gʹ* > *Gʹʹ*, the material is considered to be a solid. In the contrary case (*Gʹʹ* > *Gʹ*), the material is considered to be a liquid. Therefore, the SOD1 hydrogels are completely considered to be a solid. The properties of the SOD1 hydrogels are similar to those for a 0.25% agarose gel, showing ~300 Pa of *Gʹ* and ~10 Pa of *Gʹʹ* (S2A Fig). A shear strain sweep test showed that both the SOD1 gels in the pH 3.0 and pH 4.0 vials were stable until a 4−7% shear strain was applied (Fig 2D), which is more fragile than a 0.25% agarose gel that was stable until a 10% (S2B Fig). These results suggest that the SOD1 fibrils can form hydrogels by forming a three-dimensional cross-linked network that is held together by weak physical forces. In contrast, it was not possible to measure the *Gʹ* and *Gʹʹ* values for the pH 5.0 sample using the rheometer, because it contained numerous precipitates in the liquid and the solution too low viscoelasticity to detect *Gʹ* and *Gʹʹ*. Therefore, a suitable shear strain region for the measurement could not be found.

### QCM-A analyses distinguished the different properties of denatured SOD1 between pH 3.0, 4.0 and pH 5.0

**Fig 3:**
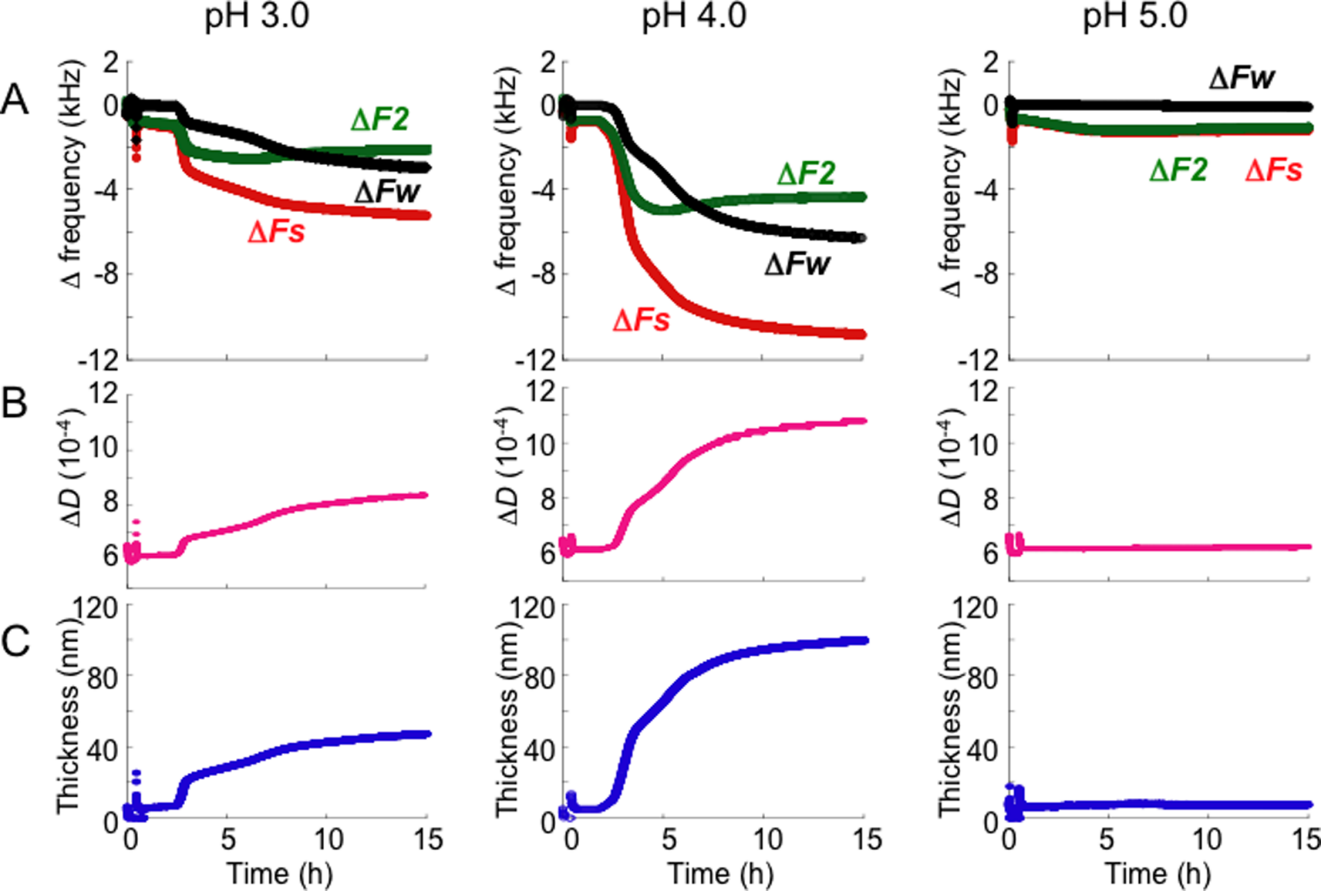
Representative raw data for three independent experiments from the QCM-A measurements upon denaturation of chemically immobilized SOD1 on the sensor, which were obtained under the fibrillation conditions in pH 3.0, 4.0 and 5.0. **(A)** Changes in three frequencies, Δ*Fs* (related to mass uptake), Δ*Fw* and Δ*F2* (related to viscoelastic properties). **(B)** Changes in the simultaneously obtained *D* values for the experiment shown in (A). **(C)** Changes in the simultaneously obtained effective acoustic thickness for the experiment shown in (A).

As described above, SOD1 fibrils progressed to form assembled forms having different properties, hydrogels at pH 3.0−4.0 or aggregates at pH 5.0 (Figs 1 and 2). In order to explore the mechanism responsible for this, we investigated the properties of the denatured form of SOD1 before self-assembly. QCM-A can measure not only changes in mass, Δ*Fs* (mainly mass, see S1 File), but also changes in energy loss (Δ*Fw*) caused by the oscillation of a material adsorbed on the electrode. In addition, −2Δ*Fw*/*Fs* is approximately equal to the energy dissipation value (*D*-value) obtained by the QCM-D method (S1 Fig). Höök et al. demonstrated that an extended flexible model protein, Mefp-1 (~22 nm thickness), resulted in decreases in the thickness (~7 nm) and the *D*-value accompanied by the release of water molecules, when it is cross-linked by the addition of NaIO_4_, by using QCM-D combined with ellipsometry and surface plasmon resonance techniques [32]. Therefore, we hypothesized that QCM-A technique can also provide viscoelastic information, such as thickness and *D*-value, on denatured SOD1 molecules prior to self-assembly to distinguish the different properties of the SOD1 samples between pH 3.0−4.0 and pH 5.0. After chemically immobilizing SOD1 on the QCM-A sensor, the final washing solution in the vessel was changed to fresh acetate buffers (pH 3.0 to 5.0) containing 10 mM EDTA and 200 mM DTT for denaturation of SOD1. Most notably, in the case of pH 4.0, clear declines in Δ*Fw* and Δ*Fs* were observed after a 2 to 3 h treatment period and reached a steady-state level after a 15 h period (Fig 3A). Since there is no SOD1 molecule in the solution after the buffer is changed, the increase in the mass (declines in Δ*Fs*) of immobilized proteins on the QCM-A sensor indicates the coupled water molecules, which could be detected even in aqueous solutions [37]. Therefore, it can be said that a lot of water molecules bound to the denatured SOD1 at pH 3.0 and 4.0 but not 5.0. Although the shift levels were small, gradual declines in Δ*Fw* and Δ*Fs* and a slight increase in Δ*F2* were also observed in the case of pH 3.0. In contrast, almost no changes in Δ*Fw*, Δ*Fs* and Δ*F2* were observed in the case of pH 5.0 until 15 h after the treatment. Changes in Δ*Fw*, Δ*Fs* and Δ*F2* after denaturation for 15 h for the three experiments are indicated in S3 Fig. Furthermore, the *D*-value (Δ*D*) (Fig 3B) and the effective acoustic thickness (Fig 3C) of the pH 3.0−4.0 adlayers simultaneously increased, but those of the pH 5.0 adlayer did not. The larger shift levels in the three frequencies and the *D*-value were observed at pH 4.0. The effective acoustic thickness of the SOD1 on the sensor at pH 3.0, 4.0 and 5.0 were 49 ± 3.9 nm, 94 ± 15 nm and 8.1 ± 0.53 nm, respectively, after denaturation for 15 h.

**Fig 4:**
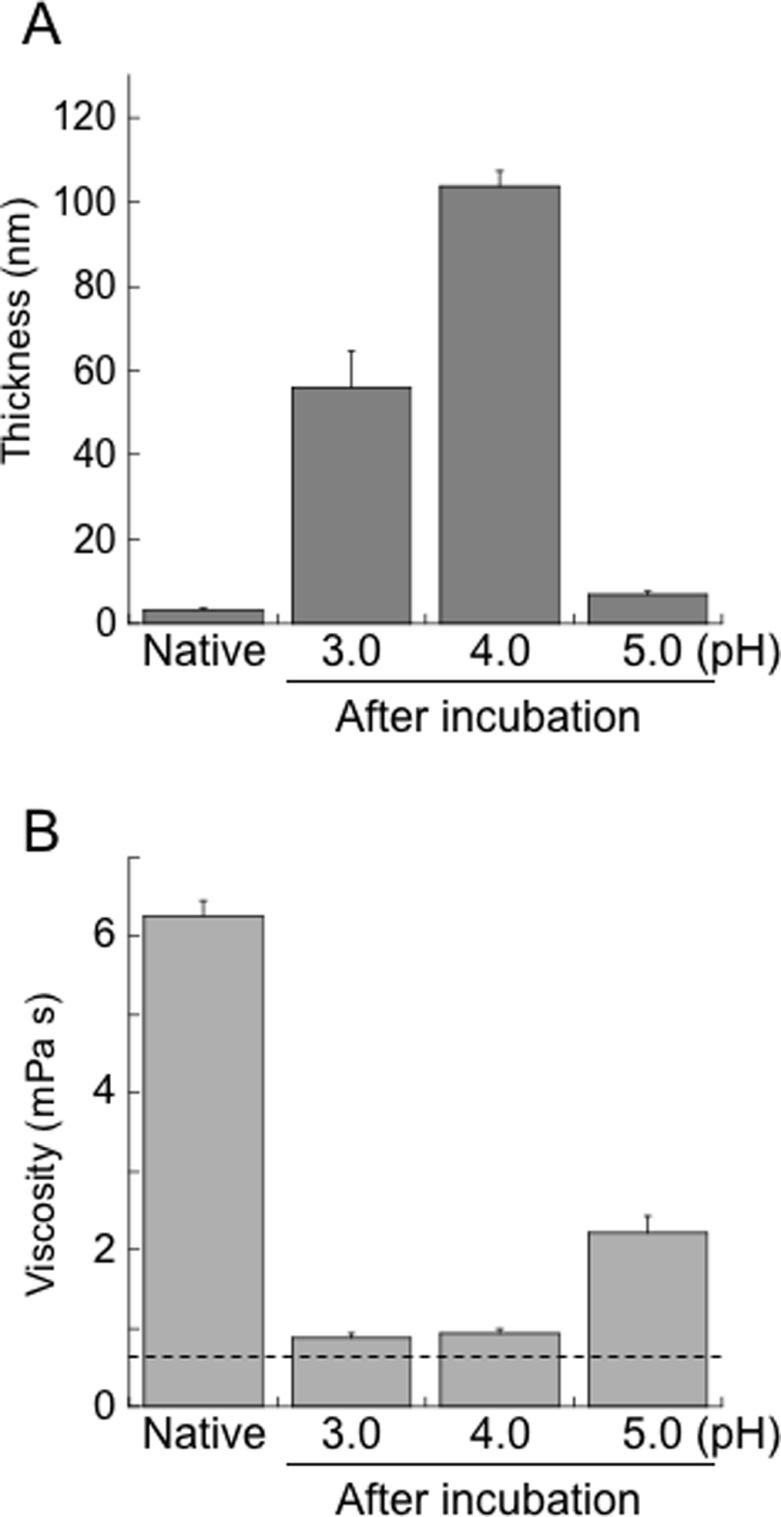
The effective acoustic thickness and the shear viscosity of directly immobilized SOD1 adlayer on a sensor. **(A)** The effective acoustic thickness of directly immobilized SOD1 adlayer on the sensor before and after denaturation at pH 3.0, 4.0 and 5.0. Data are shown as the means ± SEM from three independent experiments. (**B)** The shear viscosity of the SOD1 adlayer on the sensor before and after denaturation at pH 3.0, 4.0 and 5.0. Data are shown as the means ± SEM from three independent experiments. A dotted line means the shear viscosity of water at 37°C (0.69 mPa s).

To exclude the effect of chemical immobilizing, the direct immobilized SOD1 was next used. Similar patterns of the frequency changes as in Fig 3A were observed (data not shown), and the effective acoustic thickness of the SOD1 on the sensor at pH 3.0 and pH 4.0 also increased to 56 ± 8.6 nm and 104 ± 3.3 nm, respectively, after denaturation for 15 h from the native SOD1 state by 3.3 ± 0.16 nm (Fig 4A). Interestingly, the effective acoustic thickness of a fibrillar protein, gelatin, that was absorbed directly on the sensor was ~120 nm and those of globular proteins, IgG and BSA, were ~5 nm (S4A Fig). Furthermore, the shear viscosity of the SOD1 adlayer at both pH 3.0 and pH 4.0 after denaturation decreased to ~0.9 (0.88 ± 0.05 and 0.93 ± 0.07, respectively) mPa s from 6.9 ± 0.49 mPa s of the native SOD1 state (Fig 4B). Since the shear viscosity of pure water is 0.69 mPa s at 37°C, the viscosity of the denatured SOD1 adlayer at pH 3.0 and pH 4.0 is close to that of pure water. In contrast, the shear viscosity of the SOD1 adlayer in pH 5.0-buffer remained in the reduction of up to 2.2 ± 0.21 mPa s. Incidentally, the shear viscosity of gelatin and myosin was ~1.0 and ~1.7 mPa s, respectively, and those of globular proteins, IgG and BSA, were ~5.7 and ~5.1 mPa s, respectively (S4B Fig). These results indicate that folded globular proteins, such as SOD1, BSA and IgG, have a smaller thickness and higher viscosity. In contrast, not only fibrillar proteins like gelatin but also extended SOD1 have a larger thickness and lower viscosity.

As described above, hydrogel formation of SOD1 was strongly affected by the pH of the buffer. We also examined hydrogel formation where the pH is lower than 3.0 and pH 4.5. The SOD1 sample at pH 2 (pH 2.2, 50 mM acetic acid solution) also formed hydrogels (S5A Fig) and rapidly underwent fibrillation (S5B Fig), similar to the case of pH 3.0. In contrast, the SOD1 sample at pH 1 (pH 0.88, 50 mM phosphate solution) immediately formed a precipitate and no fibrillation was detected. At pH 4.5, although the SOD1 sample did not form a hydrogel, a slow increase in the fluorescence intensity of ThT was observed (S5 Fig). Thus, this substantial difference between pH 4.0 and 4.5 might be due to different charges on the molecule caused by acidic amino acid residues such as glutamic acid (*p*K_R_ is pH 4.25), which might affect hydrogen bonding with water molecules as well as the hydrophobicity of SOD1.

### Addition of NaCl affected fibrillation and hydrogelation

**Fig 5:**
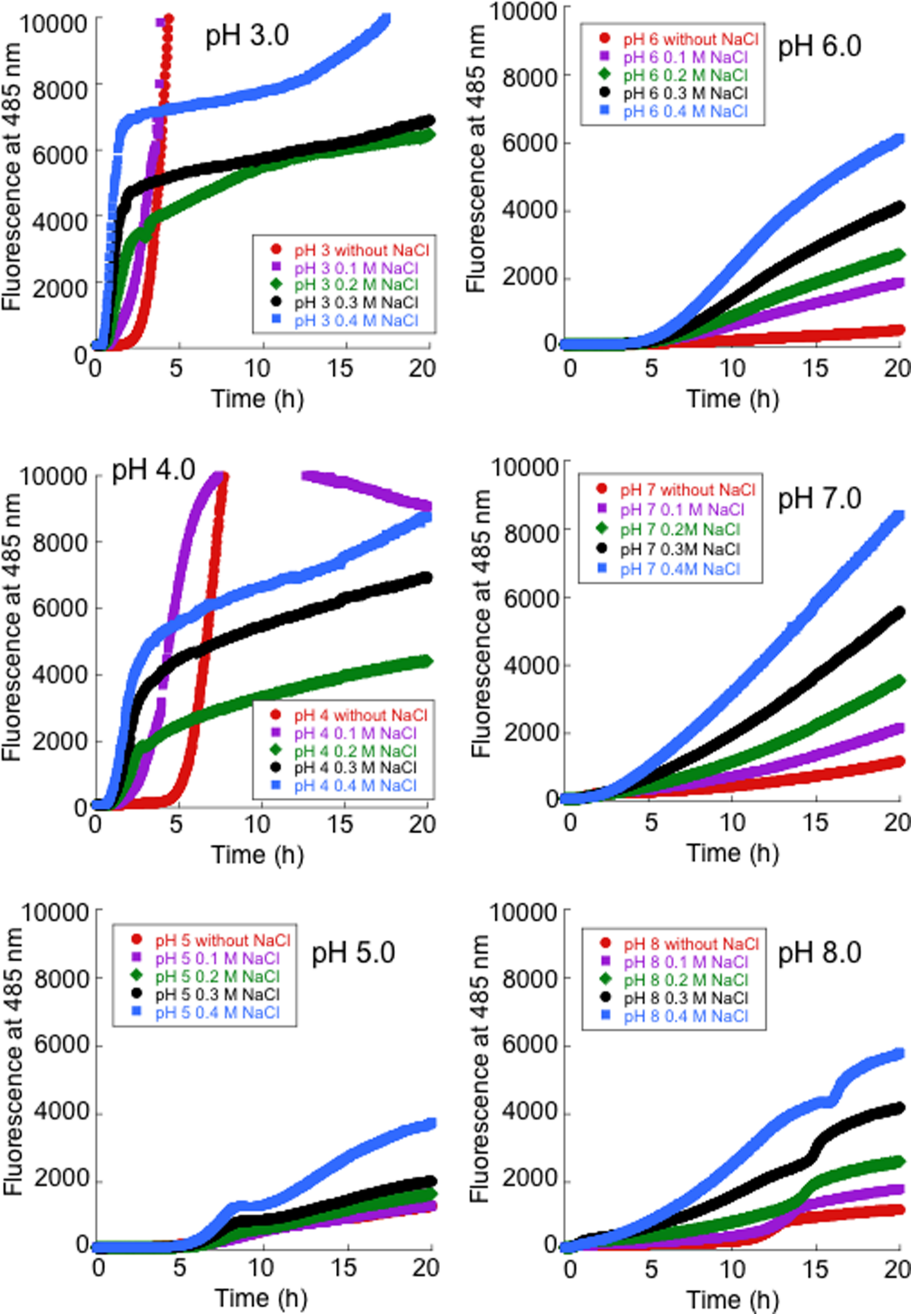
Effect of various concentrations of NaCl on SOD1 amyloid fibril growth. Fibrillation kinetics of SOD1 solutions (5 mg/mL) under fibrillation conditions in buffers at various pH values in the presence of various concentrations of NaCl, as monitored by thioflavin T fluorescence. Representative kinetic data of three ThT assays are shown.

Several reports have demonstrated that alterations in the ionic strength of the solution can influence the rate of formation of amyloid fibrils [38–40]. We therefore added various concentrations of NaCl to the ThT assay solution. As shown in Fig 5, the presence of NaCl had an accelerating effect on the initial fibrillation which increased with increasing NaCl concentration in all of the pH solutions tested. However, the rate of increase in ThT fluorescence gradually slowed when NaCl was added at pH 3.0 and 4.0. In contrast, the addition of NaCl facilitated fibrillation at pH 5.0−8.0, but the effect was minimal at pH 5.0.

**Fig 6:**
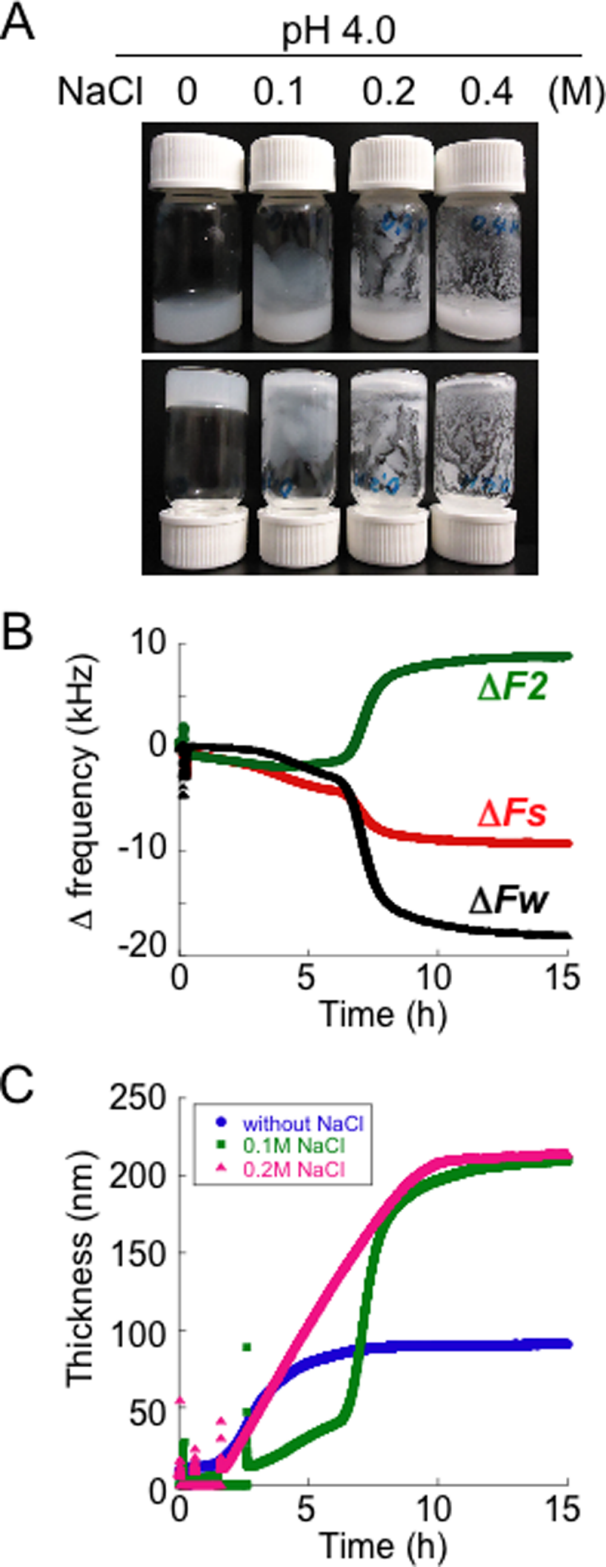
Effect of NaCl on the hydrogelation and extension of SOD1. **(A)** Images of vial inversion tests for SOD1 solutions (20 mg/mL) at pH 4.0 in the absence and presence of 0.1, 0.2 or 0.4 M NaCl after incubation at 37°C for 16 h under fibrillation conditions; upright vial bottle (upper panel) and inverted vial bottle (lower panel). **(B)** Representative raw data from three independent experiments from the QCM-A measurements upon denaturation of SOD1 that was directly immobilized on the sensor, which were obtained under the fibrillation conditions at pH 4.0 with 0.1 M NaCl. Changes in three frequencies, Δ*Fs*, Δ*Fw* and Δ*F2* (upper panel) and changes in the simultaneously obtained effective acoustic thickness (lower panel).

We next examined hydrogel formation in the presence of NaCl. As shown in Fig 6A, the addition of more than 0.1 M NaCl suppressed hydrogel formation at pH 4.0 even though rapid fibrillation was observed (Fig 5). In the presence of NaCl, these SOD1 samples formed aggregates that were similar to emulsions without visible precipitates being observed. We thus re-investigated the issue of whether the extension of SOD1 observed in the QCM-A method (Figs 3 and 4) is also suppressed by the addition of NaCl. However, a QCM-A analysis showed that the addition of 0.1 M NaCl induced further decline in Δ*Fw* and increase in Δ*F2* (Fig 6B). The effective acoustic thickness of the SOD1 on the sensor at pH 4.0 in the presence of 0.1 M NaCl was 215 ± 45.1 nm after denaturation for 15 h. We showed the representative changes in the effective acoustic thickness when 0.1 M or 0.2 M NaCl was added to the pH 4.0 sample (Fig 6C). The correct thickness of the SOD1 layer is much smaller than 200 nm as described below, but it appears that the addition of salt promotes the extension of SOD1.

In fact, the diameter of the crystal of the same protein, the 2-ME-SOD1 dimer (PDB ID 3T5W, chains A and B), is 6.8 nm (between Gly130 of both subunits) and the maximum strand length is 3.4 nm between Ala1-Asp11 in the monomer [34]. These dimensions are in reasonably good agreement with the effective acoustic thickness for the folded SOD1 (3.3 nm) that was estimated by QCM-A (Figs 3 and 4). Since the SOD1 monomer consists of 153 amino acid residues and the distance between adjacent Cα atoms is 3.8 Å in the extended states as a β-strand, the maximum length of the fully extended SOD1 monomer must be ~60 nm (578 Å). Therefore, the thicknesses detected by QCM-A, such as 100 and 200 nm (Figs 3, 4 and 6), are much larger than the actual thickness of the denatured SOD1. As proteins are denatured their structures become looser allowing them to take up more water molecules due to the increased surface area that is exposed to water [41]. Therefore, QCM-A appears to have detected an extra aqueous layer which loosely associates with the denatured SOD1 as a layer thickness. Indeed, Höök et al. compared the thickness of an adsorbed protein using ellipsometry, optical waveguide light-mode spectroscopy and QCM-D. They concluded that the mass obtained via the measured frequency shift of the QCM-D includes water in the protein layer coupled by direct hydration and/or water hydrodynamically entrapped in the adsorbed proteins whereas ellipsometry and optical waveguide light-mode spectroscopy based on optical principles did not include water molecules in the mass uptake [42]. For this reason, comparative studies with these devices or dynamic light scattering should be done in order to determine the correct thickness of the SOD1 and to investigate the relationship among the thickness, water state and the denaturation process of SOD1 on a QCM-A sensor.

Water plays an important role, not only in the structure, stability, dynamics, and function of proteins but also in unfolding and aggregation [43, 44]. Proteins are already more accessible to water even though the secondary structure is still intact at the beginning of denaturation [41]. Even in the case of native globular proteins, water molecules that are hydrogen-bonded on the protein surface are further hydrogen-bonded to form a tight water network [45]. It is therefore likely that denatured proteins form a loose larger water network on their surface. Indeed, the densities of the denatured SOD1 layer at pH 3.0 and 4.0 were calculated to be ~5 and ~10 μg/cm^2^, respectively, an increase from ~350 ng/cm^2^ for the native SOD1 that was immobilized on the sensor. It is estimated that ~1,000,000 water molecules could be coupled to one SOD1 monomer at pH 3.0. The denatured SOD1 adlayers containing such large amounts of water molecules appear to have the same viscosity as pure water (Fig 4B).

Salts also affect the charge, hydrophobicity and the conformation of the surface of proteins resulting in solubility, aggregation and fibrillation. Acceleration and the slowdown of fibril growth of *β2*-Microglobulin in the presence of lower and higher concentrations of NaCl, respectively, in acidic buffer have also been observed [39], which is consistent with our results at pH 4.0 (Fig 5). In general, salt would be expected to increase the solubility of proteins up to physiological concentrations although higher concentrations will agglutinate proteins by salting out. In a physiological concentration of a NaCl solution, sodium ions and chloride ions may form hydration shells surrounded by partially ordered water regions (outer hydration shells) by hydrogen-bonding. It is therefore possible that even larger amounts of water could associate with the denatured SOD1 in the presence of NaCl. In this case, it would not be possible to encapsulate water molecules to form a hydrogel, since the physical interaction of the SOD1 fibrils would become weak when self-assembled (Fig 6A). That is, the extent of the water network on the protein surface prior to self-assembly could affect the properties of the resulting aggregates.

Furthermore, the extent of amount of water bound to the denatured SOD1 appears to influence the rate of fibril formation. The water-poor denatured SOD1 at pH 5.0 (~10 nm) required a much longer time for fibrillation (Fig 1A). Although the effective acoustic thickness of the denatured SOD1 at pH 3.0 (~50 nm) was thinner than that at pH 4.0 (~100 nm) (Fig 4A), the rate of a fibril formation at pH 3.0 was faster than pH 4.0 (Fig 1A). At pH 4.0, addition of physiological concentrations of NaCl caused further binding of large amounts of water to the denatured SOD1 (~200 nm) (Fig 6) and a slowdown of fibril growth of SOD1 (Fig 5).

**Fig 7:**
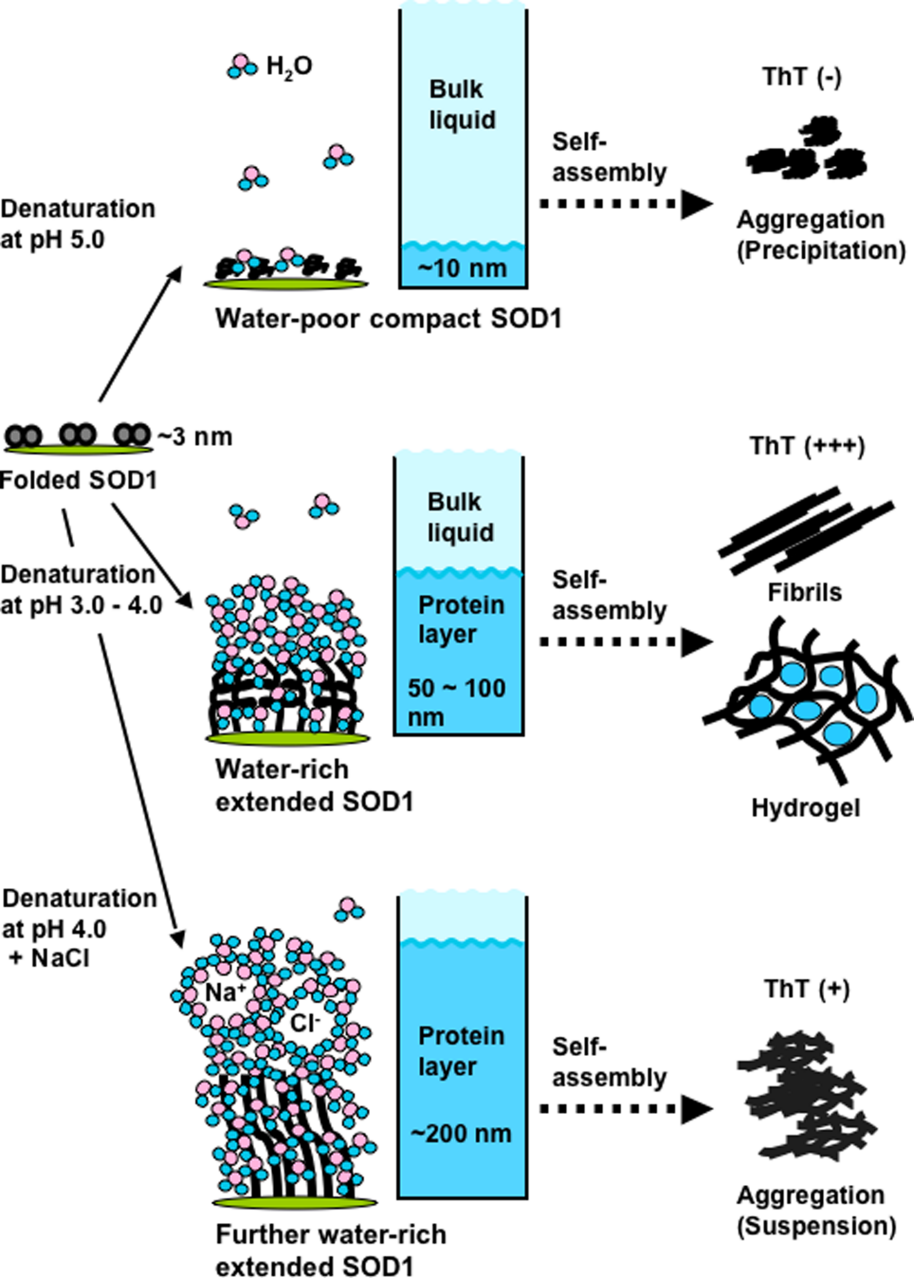
Schematic chart: Differences in the degenerative state of SOD1 result in different assembly states such as hydrogelation or precipitation.

Our findings are summarized in Fig 7. The QCM-A method could detect conformational changes of SOD1 that were accompanied by the binding and release of water molecules separately from the bulk liquid during the denaturation process. The denatured SOD1 at pH 5.0 are water-poor compact SOD1 molecules forming ThT fluorescence negative precipitates by self-assembly. In contrast, the denatured SOD1 at pH 3.0 (2.2)−4.0 was extended with large amounts of water molecules bound to them, forming ThT fluorescence positive fibrils and hydrogels by self-assembly. Moreover, when physiological concentrations of NaCl was added to the pH 4.0 sample, the SOD1 became extended with even larger amounts of water molecules bound to it and formed an aggregation-like emulsion but not a hydrogel. Therefore, a moderately water rich extended state at pH 3.0 (2.2)−4.0, which is not completely extended, would be a suitable “intermediate state” for not only hydrogelation but also for amyloid fibril formation.

According to a general model pathway, “globular proteins first unfold and lose their secondary structure, which is the intermediate state, and some of them further form amyloid fibrils and subsequently self-assemble into hydrogels” [46]. However, details of the mechanism of the hydrogelation processes are not completely understood because hydrogelation conditions are different depending on each protein or peptide. A typical hydrogel of insulin was obtained after incubation at 75°C in a pH 2 solution [47]. High concentrations of BSA can form hydrogels under both a low pH (pH 3.5) at 37°C and a neutral pH (pH 7.4) at 80°C [9]. Lysozyme can form hydrogels by preparing 43 mg/mL solution in a pH 6.9 buffer containing 20 mM DTT, heating to 85°C and then cooling to room temperature [48]. It is noteworthy that a-synuclein generates two distinct types of fibrils with normal amyloid and hydrogels, depending upon the fibrillation processes. Agitation at 200 rpm for 100 h at 37°C in 20 mM MES (pH 6.5) induced the formation of normal straight amyloid fibrils. In contrast, repeated filtrations of a-synuclein in 20 mM MES (pH 6.5) using YM-30 at 37°C induced the formation of curly amyloid fibrils and produced hydrogels composed of nano-scaled three-dimensional fibrillar networks [10]. In the case of SOD1, the intermediate state of SOD1 gel would be an extended water-rich form like gelatin (Figs 3 and 4), which would result in the rapid formation of amyloid-like fibrils (Fig 1A) and hydrogels (Figs 1 and 2).

To detect kinetics of physical gel network formation on the macro-scale, rheological measurements such as evolution of *Gʹ* and *Gʹʹ* over time, are used because rheology is considered to be the most sensitive method for material characterization compared to chemical analyses [49, 50]. Indeed, the rheological kinetic experiments showed that elongated aggregates are formed during the “apparent” lag phase of insulin amyloid gel formation [51] and the complex dynamics of gelation by islet amyloid polypeptide [5]. Thus, the measurements of viscoelastic properties of the initial assembly of SOD1 were performed. However, this rheometric method failed to reflect the different properties of the SOD1 samples between pH 3.0−4.0 and 5.0 although the values for *Gʹ* and *Gʹʹ* for all solutions immediately started to increase (S6 Fig) without a lag phase generally observed in ThT assay (Fig 1A). In addition, this measurement could not be performed until *G*ʹ would increase to ~200 Pa because it was difficult to completely prevent evaporation of the solvent more than 3 h. This first 3 h incubation might not be enough for binding of water molecules to denatured SOD1 even at pH 4.0 (Fig 3) and thus the similar property between pH 3.0, 4.0 and 5.0 would be observed. In contrast, the QCM-A analyses distinguished the different intermediate states between pH 3.0, 4.0, 5.0 and the presence of NaCl (Figs 3, 4 and 6), but they could not assess the three-dimensional assembly of denatured SOD1 because QCM-A measures properties of the thin layer adsorbed on an electrode surface.

Protein hydrogelation in vivo affects cellular functions. FUS fibrillar hydrogels showed neurotoxicity in a *C*.*elegans* model of FUS-dependent neurodegeneration by disrupting ribonucleoprotein granule function [12]. Woodard et al proposed that amyloid gels would disturb the convection for the dissolved molecules transport in the extracellular space and that TEM image of the intracellular neurofibrillary tangle composed of tau extracted from brain tissue resembled a gel [13]. Therefore, hydrogelation of amyloid like neurodegenerative proteins might be related with the diseases. ALS-linked mutant SOD1 proteins more easily form amyloid-like fibrils and aggregates compared to wild-type SOD1 [26]. Further study would be required to clarify how water molecules bind to SOD1 during unfolding and fibrillation and whether SOD1 hydrogelation links to ALS.

## Conclusion

The findings reported herein indicate, for the first time, that thioflavin T-positive fibrils of wild-type SOD1 undergoes hydrogelation at pH 3.0 (2.2)−4.0, but with precipitates being formed at pH 5.0. The QCM-A analysis showed that the immobilized SOD1 on the sensor under the hydrogelation conditions at pH 3.0−4.0 resulted in an extension accompanied by the binding of large amounts of water molecules. In contrast, at pH 5.0, the denatured SOD1 hardly bound water molecules and remained compact. On the other hand, the addition of NaCl to the pH 4.0 sample caused a further water-rich intermediate, which resulted in the formation of an aggregation like emulsion but not a hydrogel. These results suggest that different denatured intermediate states of the protein before self-assembly play a major role in determining the characteristics of the resulting aggregates, such as fibrillar hydrogels, precipitates or aggregates like emulsions. An approach using the viscoelastic properties to monitor protein unfolding and water-protein interactions would be promising in terms of developing a further understanding of protein fibrils and the development of therapeutic strategies for the treatment of neurodegenerative diseases.

## Supporting information

S1 File Theory of quartz crystal microbalance based on admittance (QCM-A) method. (PDF)

S1 Fig Overview of QCM-A method. **(A)** Conductance wave of the resonance frequency of the crystal oscillator. **(B)** Expected conductance waves and frequencies when hard materials (left panel) or soft materials (right panel) accumulated on the sensor.
(TIF)

S2 Fig Viscoelastic properties of agarose gels as determined by rheometry. **(A)** Various concentrations of agarose gels dissolved by a microwave were transferred to the stage of a rheometer. After cooling at 10°C for 10 min, storage moduli (*Gʹ*) and loss moduli (*Gʹʹ*) of the agarose gels measured at 10°C with 0.1% shear strain. **(B)** Strain dependence of *Gʹ* and *Gʹʹ* of the agarose gels measured at 10°C with an angular frequency of 20 rad/s.
(TIF)

S3 Fig Changes in Δ*Fw*, Δ*Fs* and Δ*F2* after denaturation for 15 h. Data are shown as the mean ± SEM from three independent experiments.
(TIF)

S4 Fig The effective acoustical thickness and the shear viscosity of some proteins. **(A)** The effective acoustical thickness of some proteins on the sensor immobilized directly. **(B)** The shear viscosity of some proteins on the sensor immobilized directly.
(TIF)

S5 Fig Effect of lower pH on hydogelation and fibrillation. **(A)** Images of vial inversion tests for SOD1 solutions (20 mg/mL) at pH 0.88 to 5.0 after incubation at 37°C for 16 h under fibrillation conditions; upright vial bottle (upper panel) and inverted vial bottle (lower panel). **(B)** Fibrillation kinetics of SOD1 solutions (5 mg/mL) under fibrillation conditions in buffers at pH 0.88 to 5.0, as monitored by thioflavin T fluorescence. Representative kinetic data of three ThT assays are shown.
(TIF)

S6 Fig Measurements of viscoelastic properties of the initial hydrogelation of SOD1. Rheological parameters, *Gʹ* and *Gʹʹ*, were monitored at 1 min intervals with an angular frequency of 20 rad/s at 37°C with a 5% shear strain. Initial evolution of storage moduli (*Gʹ*) (**A**) and loss moduli (*Gʹʹ*) (**B**).
(TIF)
