# Supplementary material for: Cu/Zn-superoxide dismutase forms fibrillar hydrogels in a pH-dependent manner via a water-rich extended intermediate state
Source: PLoS One. 2018 Oct 5;13(10):e0205090. doi: 10.1371/journal.pone.0205090 (PMC6173426; doi:10.1371/journal.pone.0205090)
Supplement: S1 File — (PDF) [file pone.0205090.s001.pdf]

S1 File

Theory of quartz crystal microbalance based on admittance (QCM-A) method

A 27 MHz QCM device, AFFINIX QN Pro (ULVAC, Japan), based on admittance (QCM-A) analysis [1] was used in the current study. Generally QCM detects the mass of materials that had been adsorbed to the electrode surface as a frequency change (ΔFs), which is related to mass uptake via the Sauerbrey equation [2].

$$\Delta Fs = -\frac{f_0}{\pi Z_q}\omega\rho_1 h_1$$

where ω is circular frequency (rad/s), ρ_1 is the density of the adlayer (g/cm^3), and $\omega\rho_1 h_1$ is mass load.

However, if the adsorbed layer (adlayer) on a sensor is not rigid but viscoelastic, the frequency change will not obey the Sauerbrey equation, and the change in impedance (Z (gm/sec/cm^2)) is expressed by the following equation (1) as derived by Granstaff et al [3]:

$$Z = (\omega\rho_2\eta_2/2)^{1/2}(1+j) + j\omega\rho_1\eta_1 + \frac{(G' - jG'')}{|G|^2}\omega^2\rho_2\eta_2 h_1 \text{ -------------------- (1)}$$

where ρ_2 is density of solution (g/cm^3), η_2 is the viscosity of solution (Pa s), G is the complex modulus of elasticity, G' is the storage modulus, G'' is the loss of modulus, $(\omega\rho_2\eta_2/2)^{1/2}$ is viscos load, and j is the imaginary unit.

From equation (1), the change in frequency, ΔFs, ΔFw and $\Delta F2$ would be expressed by equation (2), (3), (4), respectively.

$$\Delta Fs = -\frac{f_0}{\pi Z_q}\mathrm{Im}(Z) = -\frac{f_0}{\pi Z_q}(\omega\rho_2\eta_2/2)^{1/2} - \frac{f_0}{\pi Z_q}\omega\rho_1\eta_1 + \frac{f_0}{\pi Z_q}\frac{G''}{|G|^2}\omega^2\rho_2\eta_2 h_1 \text{ -------- (2)}$$

$$\Delta Fw = -\frac{f_0}{\pi Z_q}\mathrm{Re}(Z) = -\frac{f_0}{\pi Z_q}(\frac{\omega\rho_2\eta_2}{2})^{1/2} - \frac{f_0}{\pi Z_q}\frac{G'}{|G|^2}\omega^2\rho_2\eta_2 h_1 \quad \text{-----------------------} \quad (3)$$

$$\Delta F2 = -\frac{f_0}{\pi Z_q}\omega\rho_1 h_1 + \frac{f_0}{\pi Z_q}\frac{(G' + G'')}{|G|^2}\omega^2\rho_2\eta_2 h_1 \quad \text{--------------------------------} \quad (4)$$

, where $(\omega\rho_2\eta_2/2)^{1/2}$ is the viscous load of the solution and is regarded as 0, because we consider that there is no viscous change in the solution. The load including *G* is viscoelastic load. Because Δ*Fw* holds for only viscoelastic loads, it provides information on the viscoelastic properties of the adlayer on the sensor. In short, when Δ*Fw* is zero, the adlayer is rigid, while the adlayer is viscoelastic when Δ*Fw* is large. Δ*F2* is the frequency that is most affected by viscoelasticity and corresponds to "Δ*Fs* minus Δ*Fw*". The energy dissipation (*D*-value) is also obtained from the following equation.

$$\Delta D = -\frac{2\Delta Fw}{Fs}$$

Since the value, *Fs*, is far larger than the Δ*Fw,* Δ*D* is proportional to Δ*Fw.* Therefore, Δ*Fw* as well as Δ*D* can be an indicator for energy dissipation. Viscoelastic coefficients can be calculated from equations (2) - (4) in the fundamental wave and the third harmonic, which are in good agreement with the Voight model. The storage modulus (G'), the loss of modulus (G''), the shear viscosity (η), and the effective acoustical thickness (*h*) are also obtained.

S1(A) Fig shows the conductance wave of the resonance frequency of the crystal osillator. In this QCM-A method, three frequency parameters, Δ*Fs*, Δ*Fw* and Δ*F2*, can be monitored in a real time. When materials that are adsorbed on the sensor are "hard and rigid" such as globular proteins, Δ*Fs* and Δ*F2* would simultaneously

decrease but ΔFw would not change (S1(B) Fig, left panel). In this case, ΔFs reflects mainly mass of the materials that are adsorbed on the sensor and a decrease of 1 Hz corresponds to an increase in a mass of 0.62ng/cm^2 on the electrode. When materials that are adsorbed on the sensor are "soft and viscoelastic" such as fibrillar proteins, ΔFw would decrease and ΔFs and $\Delta F2$ are also affected by viscoelasticity, in addition to the mass. When adsorbed materials have a high viscoelasticity, $\Delta F2$ would increase (S1(B) Fig, right panel).

References

[1] Itoh, A.; Ichihashi, M. Separate measurement of the density and viscosity of a liquid using a quartz crystal microbalance based on admittance analysis (QCM-A). *Meas. Sci. Technol.* **2011**, *22*, 015402.

[2] Sauerbrey, G. Verwendung von Schwingquarzen zur Wagung dunner Schichten und zur Mikrowagung. *Z. Phys.* **1959**, *155*, 206-222.

[3] Granstaff, V.E.; Martin, J. Characterization of a Thickness-shear Mode Quartz Resonator with Multiple Nonpiezoelectric Layers. *J. Appl. Phys.* **1994**, *75*, 1319−1329.
