# Supplementary figures and images for: Cu/Zn-superoxide dismutase forms fibrillar hydrogels in a pH-dependent manner via a water-rich extended intermediate state
Source: PLoS One. 2018 Oct 5;13(10):e0205090. doi: 10.1371/journal.pone.0205090 (PMC6173426; doi:10.1371/journal.pone.0205090)

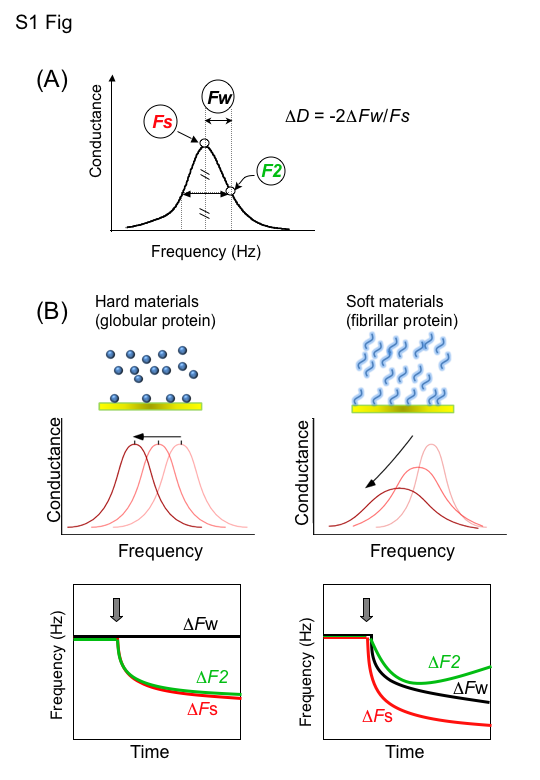

Supplement: S1 Fig — (A) Conductance wave of the resonance frequency of the crystal oscillator. (B) Expected conductance waves and frequencies when hard materials (left panel) or soft materials (right panel) accumulated on the sensor. (TIF) [file pone.0205090.s002.tif]

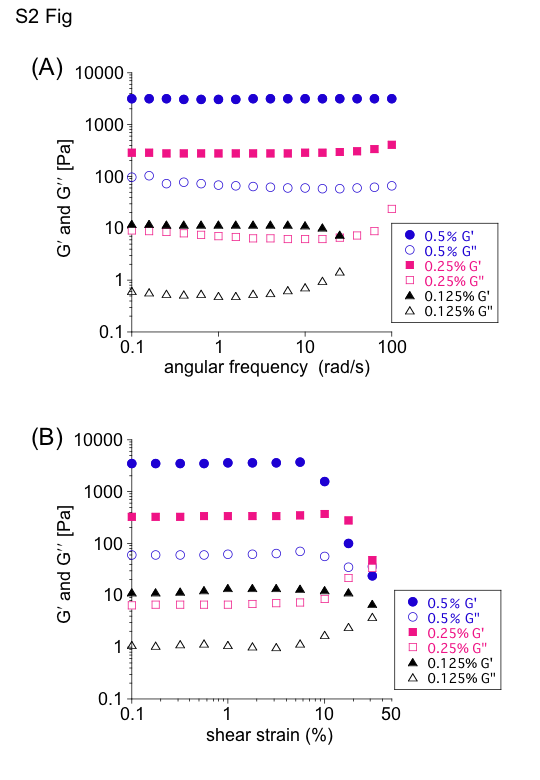

Supplement: S2 Fig — (A) Various concentrations of agarose gels dissolved by a microwave were transferred to the stage of a rheometer. After cooling at 10°C for 10 min, storage moduli (Gʹ) and loss moduli (Gʹʹ) of the agarose gels measured at 10°C with 0.1% shear strain. (B) Strain dependence of Gʹ and Gʹʹ of the agarose gels measured at 10°C with an angular frequency of 20 rad/s. (TIF) [file pone.0205090.s003.tif]

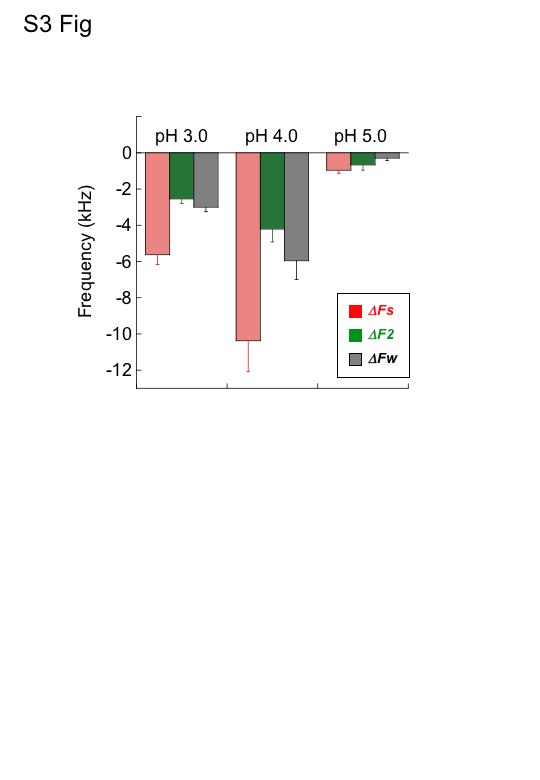

Supplement: S3 Fig — Data are shown as the mean ± SEM from three independent experiments. (TIF) [file pone.0205090.s004.tif]

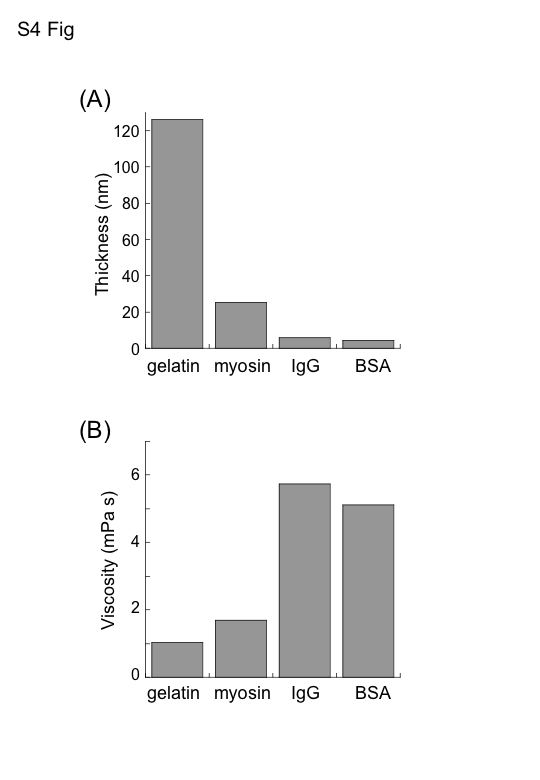

Supplement: S4 Fig — (A) The effective acoustical thickness of some proteins on the sensor immobilized directly. (B) The shear viscosity of some proteins on the sensor immobilized directly. (TIF) [file pone.0205090.s005.tif]

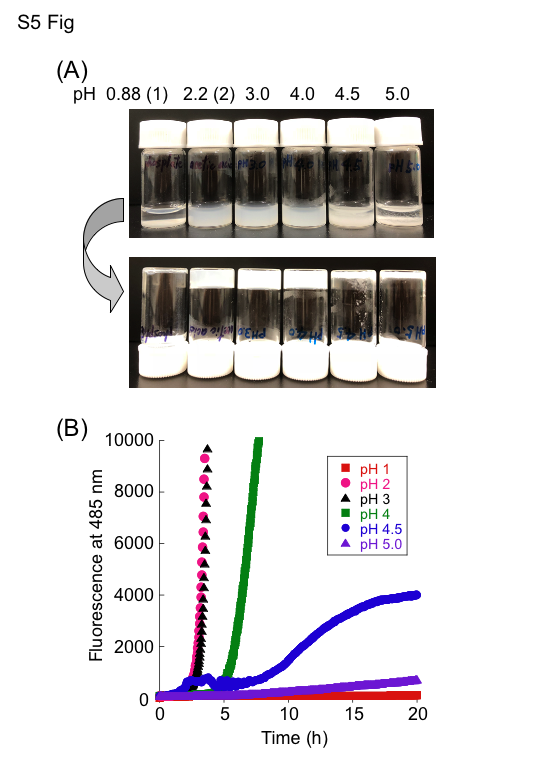

Supplement: S5 Fig — (A) Images of vial inversion tests for SOD1 solutions (20 mg/mL) at pH 0.88 to 5.0 after incubation at 37°C for 16 h under fibrillation conditions; upright vial bottle (upper panel) and inverted vial bottle (lower panel). (B) Fibrillation kinetics of SOD1 solutions (5 mg/mL) under fibrillation conditions in buffers at pH 0.88 to 5.0, as monitored by thioflavin T fluorescence. Representative kinetic data of three ThT assays are shown. (TIF) [file pone.0205090.s006.tif]

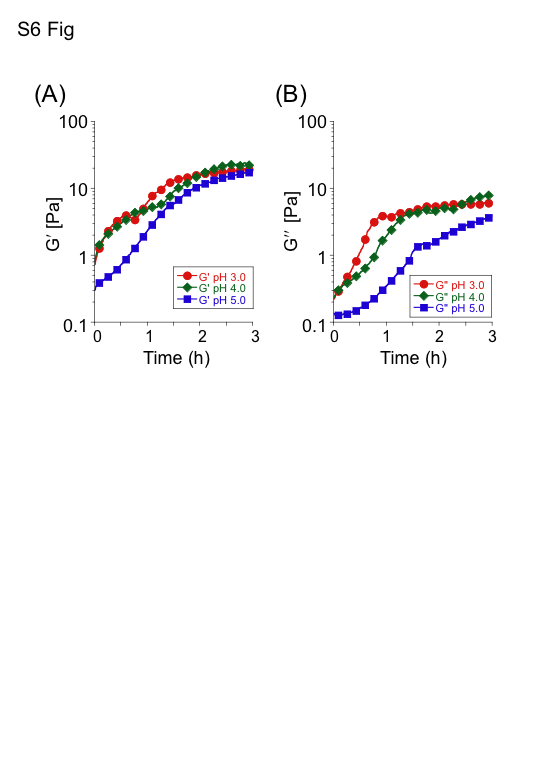

Supplement: S6 Fig — Rheological parameters, Gʹ and Gʹʹ, were monitored at 1 min intervals with an angular frequency of 20 rad/s at 37°C with a 5% shear strain. Initial evolution of storage moduli (Gʹ) (A) and loss moduli (Gʹʹ) (B). (TIF) [file pone.0205090.s007.tif]
